# Dams fed diet with no or minimal amounts of carbohydrates during pregnancy and lactation produce normal and healthy puppies: seven case reports

**DOI:** 10.3389/fvets.2026.1746349

**Published:** 2026-03-25

**Authors:** Johanna Anturaniemi, Robin Moore, Sarah Holm, Anna Hielm-Björkman

**Affiliations:** Department of Equine and Small Animal Medicine, Faculty of Veterinary Medicine, University of Helsinki, Helsinki, Finland

**Keywords:** dog, gestation, meat, processing, protein, raw diet

## Abstract

A diet containing approximately 20% carbohydrates is typically recommended for pregnant bitches. Carbohydrate-free diets have been indicated as risk factors for low birth weight, increased neonatal mortality and stillbirths, hypoglycemia, and ketosis in the dam. Here, seven cases of dams fed raw-food diets are presented. These diets contained 0 to 6% of metabolizable energy from carbohydrates. Altogether, there were 41 puppies in these seven litters, including two stillbirths and two mummified fetuses, with 100% survival among live-born puppies. Four dogs gave birth naturally, two had emergency C-sections, and one had an elective C-section. Postnatal survival compares favorably with the previously reported ranges, while prenatal loss fell within previously reported intervals. Health issues observed among the dams after giving birth included metritis in a French Bulldog and mastitis in a Mastiff. These case reports show that dams can give birth to healthy puppies with excellent survival rates, even when their diet contains few or no carbohydrates.

## Introduction

Research concerning the optimal diet during pregnancy and lactation in dogs is scarce. Compared to the maintenance levels, the protein requirements of pregnant and lactating bitches increase, but to establish more accurate protein requirements, studies using modern methodologies are needed (1). For example, a lack of protein during pregnancy can decrease the survival rate of puppies (2). Protein in the diet should primarily be of animal origin (3). In addition, fat is essential for a sufficiently energy-dense gestation and lactation diet, since it contains twice as much energy as protein or carbohydrates (4). Dietary fat supplies essential fatty acids and is necessary for the absorption of fat-soluble vitamins, and puppies can also benefit from a maternal diet rich in docosahexaenoic acid (DHA) (5, 6).

There is no minimal recommended allowance for carbohydrates in dogs (7, 8). Nevertheless, the literature often states that bitches would benefit from carbohydrates in their diet during pregnancy for optimal reproductive performance (9, 10). This assumption is primarily based on a 1981 study by Romsos et al. (9), which led to a recommendation to provide approximately 20% of metabolizable energy (ME) from carbohydrates (11).

In the aforementioned study (9), dams approximately midway through pregnancy were enrolled in a feeding study and fed either a canned diet including carbohydrates or a canned diet without carbohydrates for the remainder of the pregnancy and for 5 weeks post-weaning. Only one-third of the puppies from dams fed the carbohydrate-free diet survived 3 days after birth; half of those were stillborn. The authors concluded that the reason for stillbirth was likely the hypoglycemia observed in some bitches at whelping and that bitches therefore require carbohydrates during the final phases of gestation. Nevertheless, they also acknowledged that the dams fed the carbohydrate-free diet might have needed more protein in their diet to provide additional gluconeogenic precursors, since the amount of protein in those two diets was similar.

Subsequent studies have questioned the need for carbohydrates in pregnant bitches, although this topic has not received much attention since (12, 13). This highlights the need for updated research on the subject, since alternative diets are becoming more popular. This article presents seven case reports of dams fed raw food diets containing either no or minimal amounts of carbohydrates.

## Case presentations

We present seven cases of pregnant and lactating dams that were fed a raw food diet. The inclusion criteria stipulated that the dogs be fed raw for the entirety of their gestation and lactation. Raw feeding was chosen since the majority of raw feeders do not use carbohydrates in their dogs' diets. A raw diet was defined as feeding the pregnant dogs uncooked meat with negligible energy intake from heat-processed products. The breeders or owners provided information about the food fed to the dams during the second and 7th weeks of gestation and the 2nd week of lactation. In addition, information was requested about the number of puppies born alive or dead, birth weights, deworming, medications, type of birth, possible complications during birth, and findings in the clinical examination before rehoming. The nutritional content of the diets and calories consumed by the dams was evaluated as precisely as possible using Excel and nutritional information from manufacturers or databases such as Fineli (in Finland) (14) and the USDA (in the United States) (15). The nutritional information on the diets of all dams at all three time points is provided in Table 1, while the ingredients used are provided in Table 2.

**Table 1:** Nutritional information on the diets of seven dams during 2nd and 7th week of gestation, and 2nd week of lactation.

**Cases**	**BW kg**	**Kcal/BW^0.75^**	**Prot % ME**	**Fat % ME**	**CH % ME**	**Fiber % in DM**	**Ca % in DM**	**P % in DM**	**Vit D IU/BW^0.75^**	**Vit A IU/BW^0.75^**
Case 1: Mastiff										
Second week of gestation	80.0	147	25	74	0.3	0	2.1	1.1	157	1,321
Seventh week of gestation	83.8	154	25	73	2	0	2.0	1.1	151	1,308
Second week of lactation	80.0	223	22	76	2	0	1.8	1.0	158	1,377
Case 2: Peruvian hairless dog										
Second week of gestation	18.0	216	25	75	0	0	2.3	1.5	46^*^	320^*^
Seventh week of gestation	19.0	259	25	75	0	0	2.3	1.5	55^*^	385^*^
Second week of lactation	18.0	Fed *ad libitum*	25	75	0	0	2.3	1.5	Fed *ad libitum*	Fed *ad libitum*
Case 3: German shepherd dog										
Second week of gestation	37.0	116	26	73	1	2	3.5	1.9	13	970
Seventh week of gestation	41.9	117	29	69	1	3	3.1	1.9	41	1,991
Second week of lactation	41.9	241	30	68	1	3	3.3	2.0	94	3,604
Case 4: Shiba inu										
Second week of gestation	9.6	72	45	48	7	2	1.4	1.3	21	2,298
Seventh week of gestation	10.5	68	46	48	6	1	1.6	1.4	24	1,390
Second week of lactation	9.4	175	35	64	1	0.3	2.3	1.6	41^*^	220^*^
Case 5: Staffordshire bull terrier										
Second week of gestation	15.2	88	41	57	1	2	1.7	1.5	84	3,445
Seventh week of gestation	20.2	82	41	56	2	3	1.9	1.4	82	4,197
Second week of lactation	18.0	103	41	57	1	2	1.8	1.5	76	3,039
Case 6: French bulldog										
Second week of gestation	12.0	98	36	63	1	1	4.0	1.8	41	2,081
Seventh week of gestation	14.0	121	37	62	1	1	5.3	2.2	54	1,979
Second week of lactation	12.0	195	39	59	2	2	6.1	2.5	82	4,285
Case 7: Finnish lapphund										
Second week of gestation	16.0	103	30	68	1	2	3.0	1.8	71	1,392
Seventh week of gestation	19.0	175	32	66	1	3	3.2	1.9	101	2,130
Second week of lactation	17.0	294	30	68	1	3	3.0	1.8	184	3,272

BW, body weight; DM, dry matter; ME, metabolizable energy; Prot, protein; CH, carbohydrates; Ca, calcium; P, phosphorus.

Energy was calculated using Atwater factors.

^*^With some ingredients only the added amount by the manufacturer into the feed is known.

**Table 2:** Ingredients fed to dams during 7th week of pregnancy.

**Dam**	**Diet during 7th week of pregnancy**
Mastiff	Chicken backbones, minced beef and pork, lamb fat, beef liver, pork and chicken heart, eggs wit shells, goat milk, vitamin D supplement
Peruvian hairless dog	VOM Active Original, VOM Active salmon
Shiba Inu	Salmon filé, cooked black rice, chicken necks, goat milk, minced chicken and turkey, chicken hearts, seith, eggs, moose and beef meat, minced chicken with bone, cottage cheese, pork liver, dry food, fish oil
German shepherd dog	Mush Vaisto Puppy diets, chicken wings, eggs, liver, oils (salmon, lindseed, canola)
Staffordshire bull terrier	Chicken, beef, game and lamb meat, tripe, pork heart, meat with bone, beef internal organs, liver, fish oil, vitamin D supplement
Finnish Lapphund	Mush Duo + puppy chicken and beef, eggs, chicken necks, liver, fish, salmon oil, vitamin D supplement, kelp
French bulldog	Horse, chicken, beef and pork meat without bone, fish, turkey and reindeer meat with bone, liver, egg yolk, fish oil, kelp, folic acid, garlic

Case 1: A 5-year-old Mastiff with its first litter. The puppies were born *via* elective C-section. All three puppies were born alive. Their birth weights ranged from 498 to 605 grams. The dam had mastitis during the 2nd week of lactation, which was treated with NSAIDs and amoxicillin-clavulanic acid. None of the puppies needed any medication before rehoming. There were no abnormal findings during the puppies' health checks. During the 7th week of pregnancy, the dam's diet consisted of chicken backbones, minced beef and pork meat, lamb fat, beef liver, pork and chicken hearts, eggs with shells, goat milk, and a vitamin D supplement. The diet provided 25% ME from protein, 73% from fat, and 2% from carbohydrates. It included 6.2 g of protein per 100 kcal, which provided 9.6 g of protein per dog's metabolic weight (kg^0.75^).

Case 2: A 2-year-old Peruvian Hairless Dog with its first litter. The puppies were born via natural birth. All four puppies were born alive. Their birth weights ranged from 425 to 455 grams. The dam was dewormed once during lactation, and the puppies were dewormed at weeks four and six. One puppy was found to have a heart murmur during the health check. During the 7th week of pregnancy, the dam's diet consisted of two different commercial raw foods, marketed as complete. The diet provided 25% of ME from protein, 75% from fat, and 0% from carbohydrates. It included 6.4 g of protein per 100 kcal, which provided 16.5 g of protein/kg^0.75^.

Case 3: A 5-year-old German Shepherd Dog with its first litter. The puppies were born *via* emergency C-section, since the first puppy was found to be stuck sideways in the birth canal. Nine puppies were born alive, one was born dead, and one was mummified. Their birth weights ranged from 340 to 422 grams. The dam was dewormed once during gestation and three times during lactation, and the puppies were dewormed at weeks 3, 5, and 7. The dam received two herpes vaccinations during gestation. During the puppies' health checks, 50 % of the male puppies had not yet descended one of their testicles. During the 7th week of pregnancy, the dam's diet consisted of commercial raw diets (marketed as complete), with chicken wings, eggs, liver, and salmon and vegetable oils. The diet provided 29% of ME from protein, 69% from fat, and 1% from carbohydrates. It included 7.3 g of protein per 100 kcal, which provided 8.5 g of protein/kg^0.75^.

Case 4: A 3-year-old Shiba Inu with its first litter. The puppies were born via natural birth, and all three were born alive. Their birth weights ranged from 224 to 238 grams. The dam was dewormed three times during gestation and twice during lactation, and the puppies were dewormed at weeks 6 and 8. There were no abnormal findings during the puppies' health checks. During the 7th week of pregnancy, the dam's diet consisted of salmon filets, chicken necks, minced chicken meat with and without bones, fish, chicken hearts, pork liver, eggs, moose/turkey/beef meat, cottage cheese, goat milk, fish oil, cooked rice, and 10 grams of dry dog food per day. This diet provided 46% of ME from protein, 48% from fat, and 6% from carbohydrates. It included 11.4 g of protein per 100 kcal, which provided 7.7 g of protein/kg^0.75^.

Case 5: A 2-year-old Staffordshire Bull Terrier with its first litter. The puppies were born via natural birth, with five puppies born alive and one mummified. Their birth weights ranged from 279 to 318 grams. The dam was dewormed three times during lactation, and the puppies were dewormed at weeks 2, 4, and 6. There were no abnormal findings during the puppies' health checks. During the 7th week of pregnancy, the mother's diet consisted of chicken/beef/game/lamb meat, tripe, pork heart, meat with bones, beef organs, liver, fish oil, and a vitamin D supplement. This diet provided 41% of ME from protein, 56% from fat, and 2% from carbohydrates. It included 10.3 g of protein per 100 kcal, which provided 8.4 g of protein/kg^0.75^.

Case 6: A 4-year-old French Bulldog with its second litter. The puppies were born *via* emergency C-section, since the labor was not progressing as expected. All seven puppies were born alive. Their birth weights ranged from 160 to 243 grams. The dam was dewormed once during lactation, and the puppies were dewormed at week 5. The dam was given antibiotics after the C-section and again at 2 weeks postpartum due to metritis. The dam was vaccinated twice against the herpes virus during gestation. There were no abnormal findings during the puppies' health checks. During the 7th week of pregnancy, the dam's diet consisted of horse/chicken/beef/pork meat, fish, meat with bones, liver, egg yolk, fish oil, kelp, folic acid, and garlic. The diet provided 37% of ME from protein, 62% from fat, and 1% from carbohydrates. It included 9.2 g of protein per 100 kcal, which provided 11.1 g of protein/kg^0.75^.

Case 7: A 2-year-old Finnish Lapphund with its first litter. The puppies were born via natural birth, with six puppies born alive and one dead. Their birth weights ranged from 350 to 430 grams. The dam was dewormed once during lactation, and the puppies were dewormed at weeks 4 and 7. There were no abnormal findings during the puppies' health checks. During the 7th week of pregnancy, the diet consisted of commercial raw diets (marketed as complete), chicken hearts, eggs, fish, liver, and chicken necks. The diet provided 32% of ME from protein, 66% from fat, and 1% from carbohydrates. It included 7.9 g of protein per 100 kcal, which provided 13.8 g of protein/kg^0.75^.

## Discussion

Minimum protein requirements are typically derived from carbohydrate-containing diets. When carbohydrates are omitted, a higher dietary protein intake may be warranted to support gluconeogenesis and fetal growth (16, 17). In addition, when foods are processed, such as in canned diets, advanced glycation end products are known to be produced, which affect the bioavailability of amino acids (18).

Puppy mortality is a significant problem in canine reproduction. Studies have reported perinatal mortality rates of 2.0%−26.3%, mortality rates of 17%−30% within the first 8 weeks of life, and stillbirth rates of 2.2%−10.9% (19–23). Larger litters have been associated with a higher risk of stillbirth (19), but a recent online questionnaire study found no correlation between feeding a raw meat diet and increased puppy mortality or risk of metritis or mastitis, although the sample size was small (23). In the present series (seven litters; 41 puppies), two stillbirths (4.9%) and two mummified fetuses (4.9%) were observed, with 100% survival among live-born pups. Therefore, postnatal survival and prenatal loss lie within previously reported ranges.

### Abrupt macronutrient shifts during mid-gestation

A key contextual difference between the present report and the study by Romsos et al. (9) is the timing and stability of the maternal diet. In that study, bitches were switched at ~3.5–4 weeks post-conception to either a carbohydrate-rich diet (26% of ME from protein, 30% from fat, and 44% from carbohydrates) or a carbohydrate-free diet (26% of ME from protein, 74% from fat, and 0% from carbohydrates). Three-day postnatal survival was 93% with the mixed diet vs. 35% with the carbohydrate-free diet. In the present study, all dogs were fed raw diets before pregnancy, and the diets remained almost unchanged during gestation and lactation. Late gestation in dogs is characterized by altered basal glucose metabolism and an attenuated counter-regulatory response to hypoglycemia, which may limit short-term metabolic flexibility in the face of abrupt macronutrient changes (16, 24–26). Similar sensitivities to fuel shifts have been described in other species, such as humans and rats (27, 28). In adult, non-pregnant dogs, replacing dietary carbohydrates with fat while keeping protein low readily elevates circulating levels of the ketone body β-hydroxybutyrate, indicating that the metabolism has changed from using carbohydrates to using fat for energy (17, 29). These observations suggest that the differences in neonatal health outcomes across studies may be partly the result of different dietary structures (stable intake vs. mid-gestation shift) in pregnant bitches, whose ability to adapt to different energy sources is limited alongside their increased nutrient demands. Prospective studies that manipulate the timing and rate of macronutrient transitions are needed to evaluate this directly in bitches (16, 25, 26).

### Considerations of protein sufficiency in maternal diets

In the study by Romsos et al. (9), weekly plasma glucose and urea nitrogen did not differ between groups, although several dams on the carbohydrate-free regimen were hypoglycemic near term; both diets supplied 26% of ME from protein. In the present series, the diets provided ~25%−46% of ME from protein, with estimated protein intakes of ~7.7–16.5 g/kg^0.75^/day, compared with ~10–12 g/kg^0.75^/day in the Romsos et al. study (9). Previous studies have proposed that carbohydrate-free gestational diets should supply at least 12.7 +/– 2.08 g/kg^0.75^of protein, emphasizing both quantity and indispensable amino acid balance (12, 13).

Beyond total protein intake, protein source and digestibility may influence whether a carbohydrate-free gestational diet provides sufficient bioavailable indispensable amino acids to meet maternal and fetal requirements during pregnancy. In the carbohydrate-free formula reported by Romsos et al. (9) in Table 1, soybean protein isolate made up ~5% of the diet, while beef kidney made up ~20%. Assuming approximately 17% protein in beef kidney (15), this approximates 5 g of plant protein and 3.4–3.6 g of animal protein per 100 g of diet, or approximately a 60:40 ratio of plant to animal by mass (9). However, soy protein is limited in methionine (30), and plant proteins generally have lower digestibility (~75%−80%) than animal proteins (~90%−95%), which could alter amino acid availability when carbohydrate intake is minimal (31).

The seven cases presented here were fed raw diets that were very minimally processed compared to the canned foods used in Romsos et al. (9), which were highly processed. The very low processing of the raw diets may be one reason why these dams and their puppies were doing well, although some dams had a markedly lower protein intake per kg of metabolic weight than that proposed in earlier studies (12.7 +/– 2.08g/kg^0.75^/day) (13).

Three practical considerations emerge from prior research that may help contextualize differing observations across studies: (1) pregnancy-specific metabolic adaptations in dogs and other species may heighten sensitivity to abrupt macronutrient shifts, especially late in gestation; (2) protein quantity, e.g., ~7.7–16.5 here vs. ~10–12 or ~12.7 g/kg^0.75^/day in Romsos et al. (9) or Kienzle et al. (13) amino acid balance, and digestibility warrant particular emphasis when carbohydrates are minimized (16, 17, 25, 26, 30, 31), and (3) the amount of protein needed for optimal reproduction may differ between diets that have different processing statuses. These points are consistent with the stable diet and high survival rates of the live-born puppies observed here.

The evaluated calcium content of the diets fed to the seven bitches presented in this study varied from 1.6 to 5.3 % in dry matter (in the 7th week of gestation) and from 1.8 to 6.1 % in dry matter (in the 2nd week of lactation). The FEDIAF recommendations for minimum and maximum calcium content in reproduction diets are 1.0% and 1.6 % in dry matter, respectively (8). Diets high in calcium (2.6%−2.8 % in dry matter) have not been shown to cause adverse effects to adult dogs (32, 33), and in growing puppies, a diet containing 3.6 % of calcium in dry matter resulted in skeletal changes in beagles, but not in foxhound crossbreds (34). All of the above studies were conducted using commercial dry diets. In comparison, the natural diet of wolves has, on average, a calcium content of 1.3 % in dry matter (35), although it should be noted that wolves have a daily energy requirement that is approximately three times higher than that of adult dogs in maintenance. Nevertheless, speculation about the effects of calcium content in diets in this series of case reports is not the main focus of this study.

Pathogenic bacteria can be present in raw meat. There are a few published reports of *Campylobacter jejuni* (36), *Salmonella* spp. (37), and *Escherichia coli* (38), and abortion in dogs, and although these bacteria are not considered a common cause of pregnancy failure in dogs (39), the risk for abortion exists when the dam is fed raw meat. In addition, juvenile dogs may be more at risk for disease than adult dogs, which should be taken into consideration.

The limitation of this series of case reports is that the animals were not closely monitored, since this was not a clinical study. In addition, the breeders and owners provided the information to the authors during gestation and lactation. Therefore, the assessment of the nutritional adequacy of the provided diets was limited. Although this is paramount in clinical practice and may have been suboptimal in some cases, the focus of this series lies with the effect of carbohydrate content. Whether these diets were balanced in terms of all essential nutrients is beyond the scope of this article, but they should be critically assessed when advising owners who feed their dogs homemade diets.

Feeding of pregnant dams with no or small amounts of carbohydrates does not appear to differ from other feeding methods in terms of pup mortality or dam health, at least based on these seven cases. The diverse content suitable for carnivores and the minimal processing of proteins are likely to play an important role in assessing the necessity of carbohydrates in the diet. In the future, controlled experiments that vary both the timing of dietary transitions and the characteristics of proteins in low-carbohydrate formulations will be needed (9, 12, 13).

## Data Availability

The raw data supporting the conclusions of this article will be made available by the authors, without undue reservation.
